# Covalent Embedding of Ni^2+^/Fe^3+^ Cyanometallate Structures in Silica by Sol–Gel Processing

**DOI:** 10.1002/chem.201402805

**Published:** 2014-05-27

**Authors:** Elisabeth Felbermair, Andrey Sidorenko, Silke Paschen, Johanna Akbarzadeh, Herwig Peterlik, Ulrich Schubert

**Affiliations:** [a] Institute of Materials ChemistryVienna University of Technology, Getreidemarkt 9, 1060 Wien (Austria), Fax: (+43) 1-58801-15399; [b] Institute of Solid State Physics, Vienna University of TechnologyWiedner Hauptstrasse 8, Wien (Austria); [c] Faculty of Physics, University of ViennaBoltzmanngasse 5, 1090 Wien (Austria)

**Keywords:** cyanometallates, iron, magnetic properties, nickel, sol–gel processes

## Abstract

Compound [Ni(AEAPTS)_2_]_3_[Fe(CN)_6_]_2_ (AEAPTS=*N*-(2-aminoethyl)-3-aminopropyltrimethoxysilane), in which Ni^2+^ and Fe^3+^ ions are ferromagnetically coupled through cyano bridges, was prepared. Sol–gel processing of the AEAPTS derivative resulted in incorporation of the cyanometallate in silica. The obtained material is magnetically ordered below 22 K with an effective magnetic moment *μ*_eff_ of 4.46 μ_B_ at room temperature, a maximum of 8.60 μ_B_ at approximately 15 K and a narrow hysteresis at 2 K, with a saturation remanence of about 300 emu mol^−1^ and a coercitivity of 0.03 T.

Properties of hybrid materials can be customized by the purposeful combination of the components. This requires, among others, the deliberate selection of precursors with specified chemical composition, properties, shape and connectivity. The most common approach to hybrid materials by sol–gel processing, in which the components are connected by strong chemical bonds, is the use of molecular-precursor mixtures. An interesting alternative, which is gaining importance with the improved mastery of nano-objects, is the incorporation of pre-synthesized components, such as clusters or nanoparticles. One- or two-dimensional objects were hardly employed, but would extend the range of preparative options.

As proof of concept, we are reporting herein preliminary results on the incorporation of a cyanometallate structure into a sol–gel network. Cyanometallates often have interesting magnetic properties, because the M—N≡C—M′ bridges enable magnetic coupling between the transition metals. This could yield new property combinations for sol–gel materials. On the other hand, because the magnetic properties depend on the electronic interaction between the metals, the magnetic properties of the obtained hybrid materials can be used as for evidence whether the pre-formed structure was preserved upon sol–gel processing, which in some cases turned out to be a critical issue.

Cyanometallates form complex structures of various dimensionalities, and 0D molecular clusters,[1] 1D chains,[2] 2D sheet structures,[3] or 3D frameworks[4] have been reported. The structures and their dimensionality can be easily modified by changing the M:M′ ratio, by using different transition metals or by modifying one of the building blocks with organic ligands.[5] Changing the structure also influences the magnetic properties, which thereby are rendered tunable.[6]

One building block is typically a hexa- or octacyano-metallate anion, such as [Fe(CN)_6_]^3−^ or [W(CN)_8_]^3−^, whereas the other can be a variety of cationic transition metals or complexes. The structure and number of organic ligands on the latter influence the dimensionality of the network structure by blocking coordination sites and thus limiting the number of cyanide bridges. For example, a variety of Ni^2+^/Fe^3+^ systems with different ligands was studied by Ohba and Okawa,[7] who found structures ranging from 1D [Ni(en)_2_]_3_[Fe(CN)_6_]_2_**⋅**2 H_2_O and 2D [Ni(*N*-men)_2_]_3_-[Fe(CN)_6_]_2_**⋅***n* H_2_O to 3D [Ni(L)_2_]_3_[Fe(CN)_6_]X_2_ ((L,X)=(en, PF_6_^−^), (tn, PF_6_^−^), (tn, ClO_4_^−^); en=ethylenediamine, tn=1,3-propanediamine, *N*-men=*N*-methylethylenediamine; Scheme 1). Note that both these 1D and 2D structures have a Ni/Fe ratio of 3:2. Other examples in the Ni^2+^/Fe^3+^ system are [Ni(*N*-men)_2_]_3_[Fe(CN)_6_]_2_**⋅***n* H_2_O[7] and [Ni(cyclam)]_3_[Fe(CN)_6_]_2_**⋅***n* H_2_O (cyclam=1,4,8,11-tetraazacyclotetradecane),[8] which form honeycomb-like sheets with [Fe(CN_6_)]^3−^ units at the corners of regular hexagons. [Ni(2,3,2-tet)]_2_[Fe(CN)_6_]**⋅**8 H_2_O (2,3,2-tet=*N,N′-*bis(2-aminoethyl)-1,3-propanediamine) forms a square grid of {Ni_4_Fe_4_} units.[9]

**Scheme 1:**
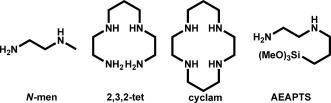
Amines used as blocking ligands.

The mentioned Ni^2+^/Fe^3+^ cyanometallate structures exhibit ferromagnetic behavior at low temperatures. Ferromagnetism in [Ni(cyclam)]_3_[Fe(CN)_6_]_2_**⋅***n* H_2_O below 8 K arises from ferromagnetic interactions between the two metals through Ni^2+^—N≡C—Fe^3+^ units with a maximum effective moment *μ*_eff_ of 11 μ_B._ The coupling between the sheets is antiferromagnetic.[8] Compound [Ni(*N*-men)_2_]_3_[Fe(CN)_6_]_2_**⋅***n* H_2_O behaves similarly with a maximum *μ*_eff_ of 25.4 μ_B_ at 7 K.[7] Ohba and Okawa reported that an anhydrous sample of the same compound is not ferromagnetic and concluded that the periodical arrangement of the sheets was destroyed.[7] A value of *μ_eff_* at room temperature below 6.3 μ_B_ was reported for both ferromagnetic structures. Complex [Ni(2,3,2-tet)]_2_[Fe(CN)_6_]**⋅**8 H_2_O is also ferromagnetic with a maximum *μ_eff_* of 4.73 μ_B_ at 6 K, which is only a small increase from the value at room temperature of 4.53 μ_B_.[9]

Herein, we focus on structures, in which [Fe(CN)_6_]^3−^ and [Ni(AEAPTS)_2_]^2+^ units (AEAPTS=*N*-(2-aminoethyl)-3-amino-propyltrimethoxysilane) are connected through Fe^3+^—C≡N—Ni^2+^ bridges. The goal was to incorporate structures similar to those described above into a silica matrix. To this end, we introduced pendant trialkoxysilyl-containing groups into the organic ligand to integrate the structures in silica through covalent bonds during sol–gel processing (Scheme 2).

**Scheme 2:**
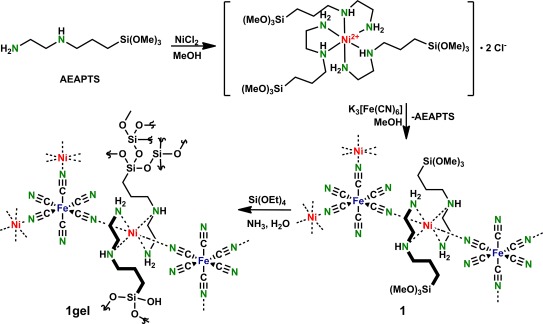
Synthesis sequence from [Ni(AEAPTS)_3_]^2+^ through the cyanometallate network to the final sol–gel material. The *trans* arrangement of the CN groups at Ni was drawn only for graphical reasons; we do not exclude the *cis* configuration.

We used *N*-(2-*a*minoethyl)-3-aminopropyltrimethoxysilane (AEAPTS) as ligand, which is similar to *N*-men, although the substituent on one of the nitrogen atoms is significantly larger. The dark violet [Ni(AEAPTS)_3_]Cl_2_,[10] obtained by addition of three molar equivalents of AEAPTS to a methanol solution of anhydrous NiCl_2_, was reacted with K_3_[Fe(CN)_6_] in methanol to give the cyanometallate [Ni(AEAPTS)_2_]_3_[Fe(CN)_6_]_2_ (**1**) as a brown powder. Sol–gel processing was subsequently performed at 70 °C by dissolving/suspending **1** in 0.2 m aqueous NH_3_ and adding tetraethoxysilane (TEOS). TEOS acts as matrix former and cross-linker. The solvent was allowed to evaporate under ambient conditions on a glass plate. The formed solid (named **1 gel**), in which **1** is tethered to the silica network by co-condensation of the Si(OMe)_3_ units of AEAPTS, was scraped off, washed with water, and dried to give a fine powder.

Each intermediate and the final product **1 gel** were analysed by FTIR spectroscopy. When going from [Ni(AEAPTS)_3_]Cl_2_ to **1**, new bands appear at 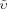
=2035 and 2106 cm^−1^ characteristic of the cyano group. The corresponding band in K_3_[Fe(CN)_6_] is at 2116; the band at 2106 cm^−1^ in **1** can therefore be assigned to non-bridging CN ligands. The additional band at 2035 cm^−1^ is due to bridging cyano groups. The main difference in the IR spectra of **1** and **1 gel** is an increase in the intensity of the broad characteristic Si—O band at 1040–1070 cm^−1^.

EDX measurement confirmed a homogeneous distribution of the metals in **1 gel**. Furthermore, a Ni/Fe ratio of about 3.3:2 was found, which corresponds quite well to the metal ratio used for the preparation of **1**. The small excess of nickel may arise from the fact that the Ni^2+^ compound is covalently linked to the silica matrix through the diamine ligand, whereas excess K_3_[Fe(CN)_6_] is not bound in the network and thus may be removed by washing with water.

Comparison of SAXS measurements of **1** and **1 gel** (Figure 1) allows determining structural changes of **1** upon embedding in silica. Compound **1** exhibits distinct Bragg reflections, which can be indexed for a hexagonal unit cell with lattice parameters *a*=*b*=1.52 nm and *c*=1.08 nm with excellent agreement between experimental and theoretical values. A structural model consistent with the SAXS data would be parallel chains arranged in a hexagonal array along *c*, in which two neighboring chains are shifted relative to each other by 0.54 nm in *c* direction, which corresponds to the size of a Ni-NC-Fe repeat unit.

**Figure 1:**
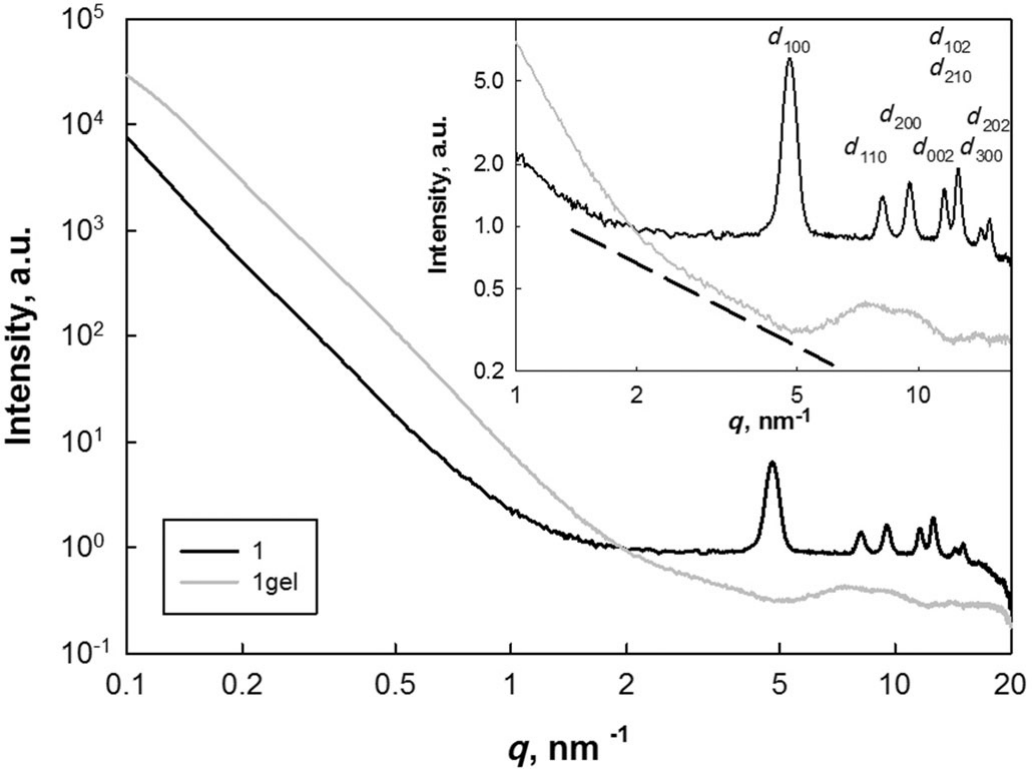
SAXS plot for 1 and 1 gel in logarithmic scale. The insert shows an enlargement of the region *q*=1–10 nm^−1^. The dashed line in the insert indicates a slope of *q*^−1^ and the reflexes of 1 are indexed according to a hexagonal lattice.

The 3D crystalline order was destroyed after sol–gel processing. The slope of *q*^−1^ (dashed line in the insert of Figure 1) between 2 and 5 nm^−1^ for **1 gel** suggests elongated objects, dispersed randomly in the silica matrix. Because the material is still ferromagnetic, this is probably due to the presence of Fe^3+^—C≡N—Ni^2+^ chains.

Magnetic measurements were performed on free-powder samples of **1 gel** on a S700X SQUID magnetometer. Field cooled (FC) and zero-field cooled (ZFC) measurements were done at different temperatures and external fields. The experimental results were corrected for diamagnetic contributions with the theoretical value calculated according to Pascal’s method.[11] Compound **1 gel** shows magnetic order below 22 K (Figure 2, insert) with signs of ferromagnetism. When plotting the molar susceptibility *χ*_m_ times the temperature (*χ*_m_**⋅***T*) versus temperature (Figure 2), an effective magnetic moment *μ*_eff_ of 4.46 *μ*_B_ (*χ*_m_**⋅***T*=*C*=2.50 emu K mol^−1^) was found at room temperature, and a maximum of 8.60 *μ*_B_ (9.25 emu K mol^−1^) at approximately 15 K. For comparison, [Ni(*N*-men)_2_]_3_[Fe(CN)_6_]_2_**⋅**12 H_2_O has a significantly higher maximum of 25.4 μ_B_ at 7.0 K,[7] but the *μ*_eff_ of 8.60 *μ*_B_ corresponds quite well to the spin-only value of 8.94 μ_B_ of two low-spin Fe^3+^ (*S*=1/2, *g*=2.00) and three Ni^2+^ (*S*=1, *g*=2.00), which are ferromagnetically coupled. A Curie temperature, *T*_C_, of 10.6 K, found for **1 gel** by fitting the magnetic susceptibility at high temperature to the Curie–Weiss law, is quite close to 10.8 K reported for bulk ferromagnetic ordering of [Ni(*N*-men)_2_]_3_[Fe(CN)_6_]_2_**⋅**12 H_2_O. The positive Curie temperature further indicates a ferromagnetic ordering of the Ni^2+^ and Fe^3+^ centres in **1 gel**.

**Figure 2:**
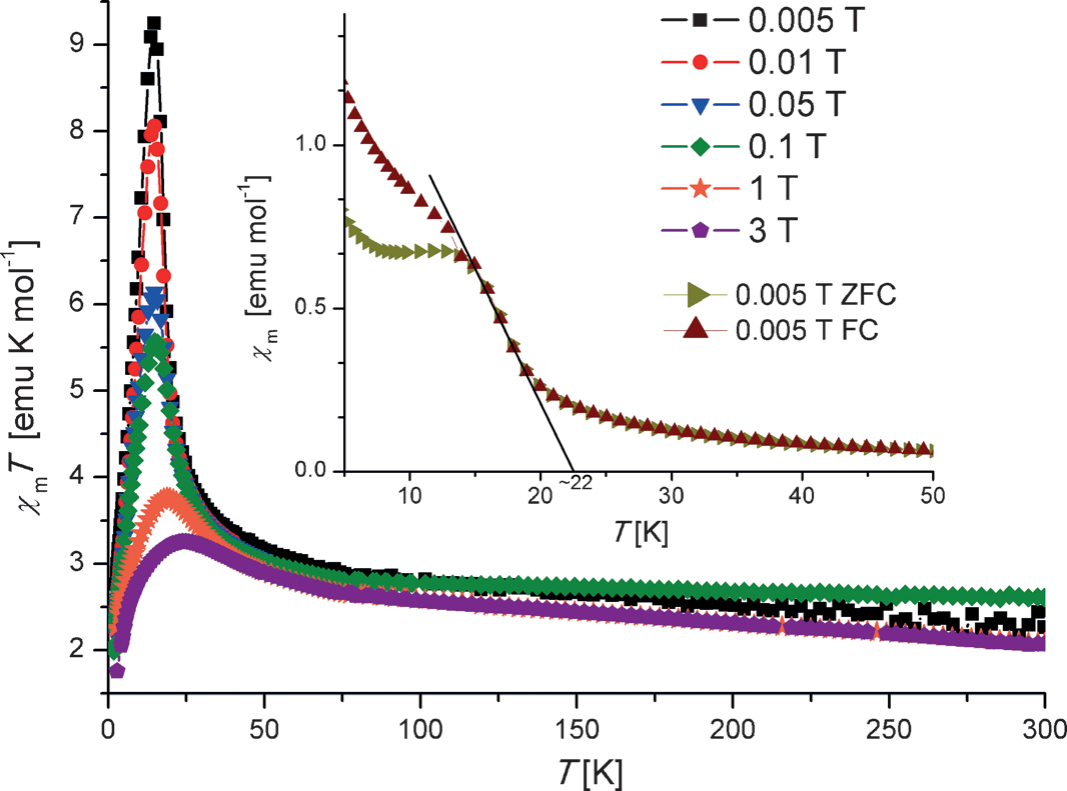
SQUID molar susceptibility times temperature (*χ*_m_⋅*T*) versus temperature (*T*) at various fields and molar susceptibility *χ*_m_ versus temperature *T* (insert) at an external field of 0.005 T (zero field cooled (ZFC), field cooled (FC)) plots for 1 gel.

Additional evidence for ferromagnetic order of **1 gel** was found in a magnetisation *M* versus applied magnetic field *μ*_0_*H* measurement at different temperatures. The hysteresis is narrow at approximately 2 K (Figure 3), with a remanence magnetisation of about 300 emu mol^−1^ and a coercitivity of 0.03 T. The hysteresis narrows with increasing temperature and collapses at high temperature, when **1 gel** becomes paramagnetic and exhibits a linear magnetic-field dependence of magnetism.

**Figure 3:**
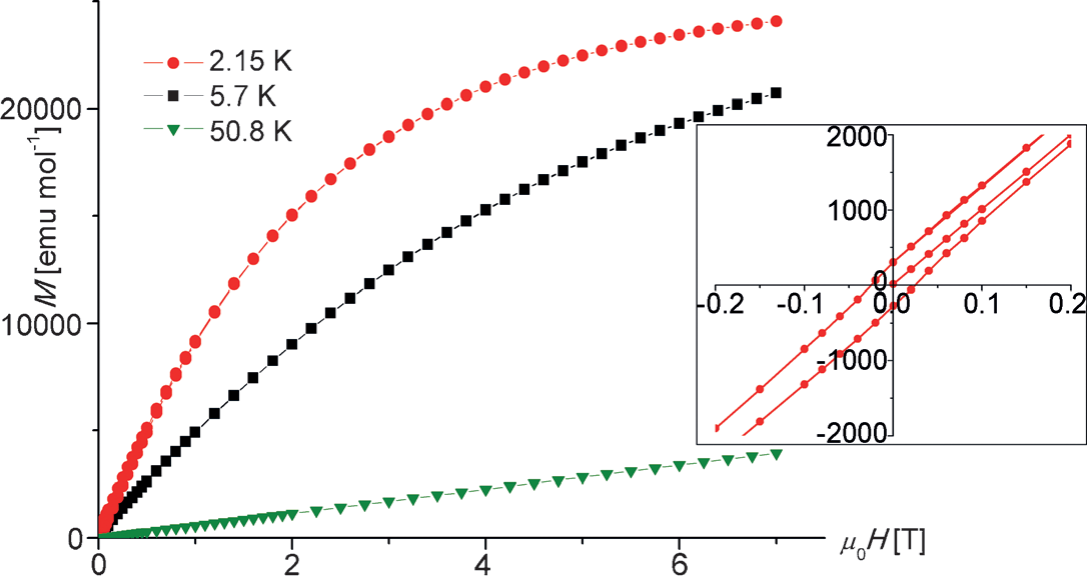
SQUID magnetization *M* versus external magnetic field *μ*_0_*H* plot for 1 gel. The insert shows an enlargement for the measurement at 2.15 K.

In summary, we have synthesized [Ni(AEAPTS)_2_]_3_[Fe(CN)_6_]_2_ (**1**), which was then covalently embedded in silica (**1 gel**). AEAPTS acts as both a blocking ligand at Ni^2+^ and as a linker to tether the cyanometallate structure to the silica matrix during sol–gel processing through reaction of the trialkoxysilyl groups. SQUID measurements showed that **1a** exhibits magnetic ordering below 22 K with an effective magnetic moment *μ_eff_* of 4.46 μ_B_ at room temperature and a maximum of 8.60 μ_B_ at approximately 15 K. Combination with FTIR, EDX, and SAXS measurements led to the conclusion that cyanometallate structures are retained in the gel, even if structural alignment in the third dimension is disrupted.

## Experimental Section

### General methods and materials

Most reagents were obtained from commercial sources and used without further purification. NiCl_2_**⋅**6 H_2_O was dried at 120 °C to obtain yellow anhydrous NiCl_2_. Solvents were dried by standard techniques and stored over molecular sieves under argon atmosphere. FTIR spectra were recorded on a Bruker IR Tensor 27 in ATR.

### Magnetic measurements

Magnetic properties were measured on a S700X SQUID magnetometer (Cryogenic Ltd.) on free-powder samples in a gelatin capsule. The magnetic moments were corrected for the diamagnetic background of the capsule. Zero field cooled (ZFC) and field cooled (FC) data were collected in the temperature range 2–300 K with various applied fields. The measurements of *M* versus *μ*_0_*H* curves at different temperatures were carried out after cooling the samples in zero field. The obtained results were corrected for diamagnetic contributions of the sample by subtraction of theoretical values obtained according to Pascal’s method. The effective magnetic moments were calculated according to the equation *μ*_eff_=2.828(*χ*_m_**⋅***T*)^1/2^.

### SAXS

Small- and wide-angle experiments were performed with a Bruker Nanostar, equipped with a 2D-position sensitive detector (Vantec 2000). The scattering patterns were taken typically for 600 s. The radially integrated data were corrected for background scattering, small- and wide-angle data were merged together to result in scattering intensities in dependence on the scattering vector *q*=(4*π*/*λ*) sin *θ* in the range from *q*=0.1 to 20 nm^−1^, in which 2*θ* is the scattering angle and *λ*=0.1542 nm the X-ray wavelength.

### Synthesis of [Ni(AEAPTS)_2_]_3_[Fe(CN)_6_]_2_ (1)

A heated (50 °C) solution of K_3_[Fe(CN)_6_] (2 equiv) in 65 mL mmol^−1^ of dry methanol was slowly added to a heated solution of [Ni(AEAPTS)_3_]Cl_2_[10] (3 equiv) in 40 mL mmol^−1^ of dry methanol. A precipitate was formed immediately and filtered off, washed with dry ethanol, and dried in vacuum to obtain **1** in quantitate yield.

### Sol–gel processing

Compound **1** (1 equiv) and tetraethoxysilane (2 equiv) were dispersed in 330 mL mg^−1^ **1** of 0.2 m NH_3_ and stirred for 2 d at 70 °C. The reaction solution was cooled to room temperature and then poured on a glass plate. The resulting solid (**1 gel**), obtained as a fine powder in quantitate yield, was then scraped off.
